# Fluopyram in agricultural ecosystems: environmental fate, microbial transformation, ecotoxicity, and resistance risks of an SDHI fungicide

**DOI:** 10.3389/fmicb.2026.1890957

**Published:** 2026-09-03

**Authors:** Chaoqun Zhang, Muhammad Zafar Ullah, Chengwei Guan, Mustansar Mubeen, Yongliang Ding, Yasir Iftikhar, Wenmei Zhang, Muhammad Asam Riaz, Muhammad Tanveer Altaf, Ahmed Mahmoud Ismail, Brijendra Kumar Kashyap, Manoj Kumar Solanki, Jie Wang

**Affiliations:** 1 Jiangxi Tobacco Science Research Institute, Nanchang, China; 2 Centre of Excellence for Citrus, College of Agriculture, University of Sargodha, Sargodha, Pakistan; 3 School of Life Sciences and Medical Engineering, Anhui University, Hefei, China; 4 Department of Plant Pathology, College of Agriculture, University of Sargodha, Sargodha, Pakistan; 5 Department of Entomology, College of Agriculture, University of Sargodha, Sargodha, Pakistan; 6 Department of Field Crops, Faculty of Agriculture, Recep Tayyip Erdogan University, Rize/Pazar, Türkiye; 7 Pests and Plant Diseases Unit, College of Agricultural and Food Sciences, King Faisal University, Al-Ahsa, Saudi Arabia; 8 Department of Biotechnology, Institute of Engineering and Technology, Bundelkhand University, Jhansi, Uttar Pradesh, India; 9 Department of Life Sciences and Biological Sciences, IES University, Bhopal, Madhya Pradesh, India; 10 Tobacco Research Institute, Chinese Academy of Agricultural Sciences, Qingdao, China

**Keywords:** ecotoxicity, environmental microbiology, fluopyram, fungicide resistance, microbial transformation, SDHI fungicide

## Abstract

Fluopyram is a next-generation succinate dehydrogenase inhibitor (SDHI) fungicide, which is very popular in the management of economically significant fungal conditions in cereals, fruits, vegetables, and ornamental crops. It works by inhibiting the complex II present in the mitochondria by blocking succinate dehydrogenase, which electron transport and energy generation, eventually hindering fungal development leading to death of the pathogen. Besides having good fungicidal effects, fluopyram has also demonstrated a nematicidal effect, which has also enhanced its utility in various agricultural systems. Empirical studies indicate that SDHIs can induce mortality, mitochondrial dysfunction, oxidative stress, and developmental delays in non-target organisms. Additionally, the environmental persistence of these compounds raises concerns about their potential for ecological disruption. The extensive and growing use of SDHIs however has also given rise to the issue of resistance development with recorded resistance cases in a number of fungal populations and has placed a strong demand on the need to use crop rotation strategies, combination formulations and resistance monitoring initiatives. In addition to its agronomic advantages, the environmental fate of fluopyram such as its mobility, persistence and degradation characteristics in soil and water and the possibility of food system residues have been the subject of attention. Unlike previous studies focusing on isolated aspects, this review comprehensively links efficacy, environmental dynamics, and mechanistic toxicity of fluopyram across multiple biological systems. It highlights underexplored areas including metabolite toxicity, chronic ecological exposure, and resistance risks. This integrated approach offers valuable insights for safer pesticide management and future research directions.

## 1 Introduction

**Figure 1:**
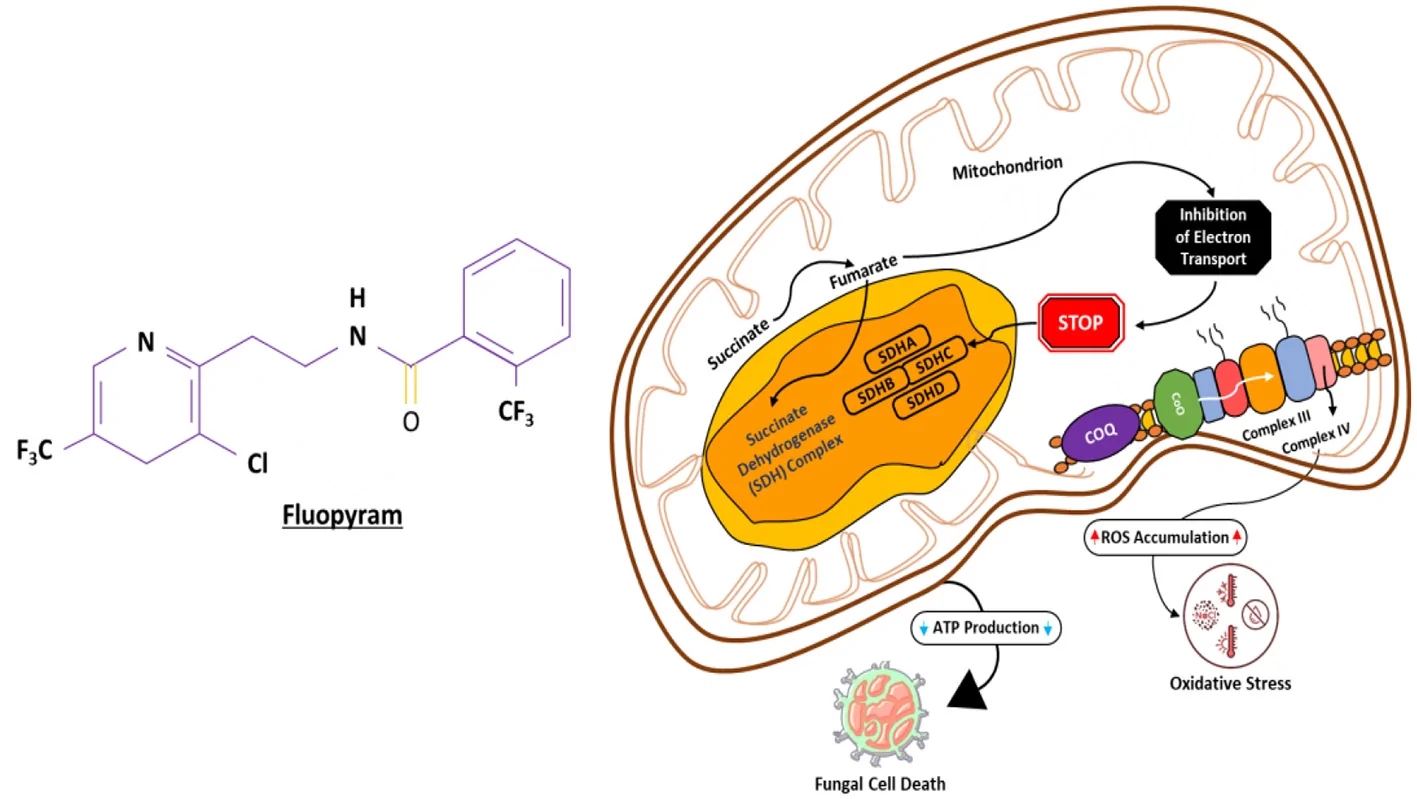
Chemical structure and SDH inhibition pathway of fluopyram.

The development of fluopyram is a prime example of innovation in agriculture by combining both fungicidal and nematicidal activity in a single product, allowing for the effective control of multiple soil-borne pathogens and plant-parasitic nematodes (PPN) in a single application (Rocha et al., 2022). Fluopyram is now employed in a variety of crops, such as soybeans, tomatoes, rice, wheat, strawberries, eggplant and peppers, and it is known to promote plant growth, lower disease incidence, and maintain plant health (Ji et al., 2019; Haque and Khan, 2022; Xu et al., 2025). Fluopyram also can produce direct pesticidal activity and have a positive effect on beneficial soil microorganisms, which in turn can lead to enhanced plant growth and nutrient use efficiency (Rocha et al., 2022). The earlier SDHI fungicides were effective against fungal pathogens, but they possessed very limited activity, were reported to be facing resistance and had inconsistent efficacy in field conditions which forced the development of newer molecules with better properties (Rathod et al., 2022). Fluopyram was developed as a second-generation SDHI with a combination of broad spectrum biological activity, plant systemic activity and nematicidal activity (Schleker et al., 2022). In comparison to previous SDHIs, Fluopyram has a higher binding affinity for succinate dehydrogenase enzymes and greater persistence, which can provide a longer protection against target organisms (Schleker et al., 2022). Second-generation succinate dehydrogenase inhibitors (SDHIs) are the promising type of the fungicides because of their distinct chemical structures, excellent biological properties, and wide-spectrum activity against phytopathogenic fungi, which can be used as the future candidates of development of fungicides (Li et al., 2021). Fluensulfone, fluopyram and fluazaindolizine are examples of non-fumigant nematicides that offer efficient control of nematodes, but they have relatively low toxicity levels in non-target organisms and thus can be used in intensive agricultural systems (Oka, 2020). The new subclass of SDHI is isofluencypram, an agent with long-term activity against major cereal foliar diseases when interacting with the ubiquinone binding site of the enzyme through a non-categorical interaction (Desbordes et al., 2020). Fluopyram is a non-fumigant nematicide with high lipophilicity and stability, which makes it persist longer in soil and sediment environments compared to many other SDHI fungicides with shorter environmental half-lives (Rathod et al., 2022). The ability of fluopyram to inhibit succinate dehydrogenase enzyme in the fungal mitochondria disrupts cellular respiration and makes it a globally significant SDHI fungicide-nematicide because it can be used against fungi and plant-parasitic nematodes, therefore, reducing the need to use multiple pesticides to achieve pest control and lowering the overall costs of crop protection (Ji et al., 2019; Haque and Khan, 2022). Human contact with fluopyram mainly happens in dietary consumption of treated crops, in workplaces in the pesticides application, and in the natural environment through the polluted soil and water (Rathod et al., 2022). SDH inhibition leads to the accumulation of succinate in the intracellular environment disrupting the homeostasis of metabolism and cellular communication (Dalla Pozza et al., 2020). SDH quantifies in plant pathogens cause stagnation of growth, delayed spore germination, and cell death, which makes SDHIs an excellent fungicide (He et al., 2023). The inhibition of SDH in plant-parasitic nematodes decreases motility, infectivity and survival by reducing the availability of energy (Schleker et al., 2022). SDH is a highly conserved evolutionary protein, which can have biological effects in fungi, nematodes, animals, and humans because of its high levels of conservation (Bénit et al., 2019). Fluopyram is a multi-action fungicide and plant-parasitic nematode that provides a number of agronomic benefits as it controls both fungal diseases and plant-parasitic nematodes in the same application, thus minimizing crop losses and maximizing productivity (Haque and Khan, 2022). It may improve the growth and yield of some plants such as soybean, tomato, pepper and strawberry (Ji et al., 2019; Rocha et al., 2022). Fluopyram seed treatments can be applied to provide protection during early season without compromising seed germination or crop establishment (Xu et al., 2025). The SDH inhibition has clinical relevance with it being an important hallmark of multiple cancers where loss or depletion of SDH function is observed, such as renal cell carcinoma and paraganglioma (Gill, 2018; Aggarwal et al., 2021). The chemical structure of fluopyram and how it works as a succinate dehydrogenase inhibitor (SDHI). Fluopyram inhibits electron transfer from succinate to ubiquinone in the mitochondrial electron transport chain by binding to succinate dehydrogenase (Complex II). This inhibition leads to diminished mitochondrial respiration and ATP production, leading to energy deficiency in the cell. At the same time, reduced electron transport causes the buildup of reactive oxygen species (ROS) which causes oxidative stress and damage to cells (Figure 1). Taken together, these impacts negatively affect the metabolic functions and ultimately lead to the death of fungal cells (Avenot and Michailides, 2010; Sierotzki and Scalliet, 2013).

### 1.2 Supporting methodology

The fluopyram was carried out by structured survey method by collecting peer-reviewed articles from several scientific databases e.g., Sciencedirect, Web of Science, PubMed, Scopus and Google Scholar. The previous studies conducted on the fluopyram had applied the similar data-based approach of database-driven residue analysis, environmental behavior and toxicological assessment so that sufficient coverage of the content subject can be discharged (Vargas-Pérez et al., 2020; Ren et al., 2023). Previous researches (2016–2020) had predominantly investigated the degradation behavior of fluopyram, the behavior of residues, and their persistence in the field through field experiments and chromatographic analysis methods (Wei et al., 2016; Dong and Hu, 2016). Furthermore, recent studies (2021–2026) have utilized cutting-edge analytical and instrumental methods, like UHPLC-MS/MS, voltammetry, immunoassays, and the strategies of nano-formulations to enhance the detection of these compounds as well as their efficacy evaluation (Rajput et al., 2018; Wang et al., 2023; Levent, 2026). In the analytical and environmental areas, researchers have applied multi-matrix sampling (soil, water, food, and biological systems), along with the use of state-of-the-art instrumental methods, to evaluate fluopyram residues and the toxicity of its presence (Silva et al., 2023; Zhou et al., 2025). Only peer-reviewed studies published in the last 10 years (2016–2026) were used to ensure both basic and latest research in fluopyram was involved (Table 1). The environmental fate of fluopyram is strongly influenced by its low water solubility, moderate lipophilicity, and high soil adsorption characteristics (Table 2).

**Table 1:** Comparative overview of fluopyram and other commonly used SDHI fungicides (boscalid, fluxapyroxad, and pydiflumetofen) highlighting their chemical class, spectrum of activity, environmental persistence, and resistance risk.

Feature	Fluopyram	Boscalid	Fluxapyroxad	Pydiflumetofen	References
Chemical class/generation	SDHI with dual fungicide + nematicide activity	First-generation SDHI fungicide	New-generation SDHI with broad-spectrum activity	Advanced SDHI with high potency and lipophilicity	Sierotzki and Scalliet, 2013
Mode of action	Inhibits mitochondrial complex II (SDH enzyme) in fungi & nematodes	SDH inhibition at ubiquinone binding site	Blocks electron transport in fungal mitochondria	Strong inhibition of SDH leading to energy disruption	FRAC, 2021
Environmental persistence	High soil adsorption and long persistence (up to ~347 days)	Moderate persistence (~200 days range)	Moderate–high persistence in soil & water systems	High lipophilicity with environmental persistence concerns	European Food Safety Authority (EFSA) et al., 2020; Zhang et al., 2024; Yanicostas and Soussi-Yanicostas, 2021
Ecotoxicity/resistance risk	Moderate ecological risk + nematicidal use increases exposure routes	Moderate resistance risk in field fungi	Higher aquatic toxicity potential in sensitive species	High resistance risk due to SDHI cross-resistance	Fernández-Ortuão et al., 2017

**Table 2:** Key physicochemical properties of fluopyram that govern its environmental fate, including water solubility, lipophilicity, soil adsorption, and persistence under different environmental conditions.

Property	Description	References
Water solubility	Low solubility (~1 mg/L−5 mg/L), limits mobility in water systems	Vargas-Pérez et al., 2020
Lipophilicity (Log Kow)	Moderately high (~3.3–3.5), promotes adsorption to organic matter	Zhou et al., 2021
Soil adsorption (Koc)	High adsorption to soil particles, low leaching potential	Wei et al., 2016
Environmental persistence	Moderate to high half-life depending on soil and climate conditions	Paszko et al., 2025

## 2 Agricultural efficacy and spectrum of activity

SDHI fungicides have been sold as single active ingredient or in combination with other fungicides with different modes of action to enhance efficacy and extend field life (FRAC, 2024). Single-active products offer targeted disease control, long residual activity and high activity against many fungus and plant-parasitic nematodes pathogens (Veloukas and Karaoglanidis, 2012; Ji et al., 2019). A number of SDHI fungicides are commercially available as a combination fungicide to enhance disease control and minimize the risk of resistance (FRAC, 2024). Fluopyram is often used in combination with a quinone outside inhibitor (QoI) fungicide as the two have different mechanisms of fungal respiration (Duan et al., 2019). SDHI fungicides are highly effective against *Botrytis cinerea* that causes gray mold in grapevine, strawberry, tomato, and ornamental crops, and are therefore useful in the high-value horticultural crops (Ishii et al., 2016; Du et al., 2025). In rice cultivation, SDHI fungicides are used to manage *Fusarium fujikuroi* which causes bakanae disease in rice, resulting in better seedling growth and yield stability (Sun et al., 2021). New conjugated alkyl SDHI compounds exhibit high activity in the treatment of *Phytophthora infestans* and *Pythium* spp., which will results in the management of late blight and damping-off diseases in potato and vegetable crops (Bao et al., 2025). Fluopyrams with guanidine groups have an improved antifungal activity against Fungi, *Sclerotinia sclerotiorum*, and *Fusarium* spp., which expands its use in fruits, vegetables, and field crops (Liang et al., 2021). Some SDHI fungicides, such as benzovindiflupyr, pydiflumetofen, and derivatives of pyrazole-benzamide, are characterized by a high preventive effect, as they inhibit the early infection of the fungus and growth of mycelia (Ishii et al., 2016; Duan et al., 2019; Li et al., 2025). Some new SDHI derivatives are also curatively effective in the control of disease progression following infection, and thus have dual protective and therapeutic promise in the management of crop disease (Gao et al., 2020; Du et al., 2025). The variation in binding interactions between SDHI fungicides and the SDH complex determines the antifungal activity and the risk of resistance, and the necessity of the use of resistance-management strategies and structural diversification (Gao et al., 2020). SDHI-based nematicides have similar or even better nematicide control compared to the traditional nematicides and less environmental persistence and reduced application rate (Liu et al., 2024). SDHIs are dual fungicidal-nematicidal agents, which is why they are an attractive alternative to the traditional single-target nematicides in integrated pest management systems (Liang et al., 2021). The combination of fluopyram with QoI fungicides like trifloxystrobin applied in synergetic formulations results in better disease management through acting on more than one of the fungal metabolic pathways, which increases the spectrum and longevity of the activity (Duan et al., 2019). The variety of complaints from growers about failures of one fungicide to manage all pathogens led them to develop mixed-active formulations to extend the spectrum of activity (FRAC, 2024). The use of so-called “rotational strategy” (combination of SDHI and fungicides with different modes of action) leads to more biochemical targets within the pathogen and more disease suppression (Duan et al., 2019).

### 2.1 FRAC-based resistance risk associated with SDHI fungicides

Based on the Fungicide Resistance Action Committee (FRAC), SDHI fungicides fell into FRAC Group 7 defined as fungicides with a medium to high risk of development of resistance due to targeting a single biochemical site (FRAC, 2024). SDHI fungicides are inhibitors of succinate dehydrogenase (SDH) in mitochondrial complex II, and mutations in the SDH enzyme can have a major impact on fungicide susceptibility (Gao et al., 2020). FRAC recommends reducing the total number of SDHI applications during the growing season, and space the use with SDHI products of the same mode of action (MOA) as much as possible (Table 3) (FRAC, 2024).

**Table 3:** The distinguishing characteristics of SDHI fungicides and fluopyram.

Feature	SDHIs (general class)	Fluopyram (specific compound)	References
Definition	Class of fungicides targeting succinate dehydrogenase (complex II) in mitochondria	A specific SDHI fungicide with nematicidal and fungicidal activity	Bénit et al., 2022
Mode of action	Inhibit SDH enzyme leading to disrupted mitochondrial respiration	Specifically binds SDH enzyme causing inhibition in fungi and nematodes	Bouillaud, 2023; Ji et al., 2019
Scope of activity	Broad SDHI activity across multiple compounds (e.g., boscalid, fluxapyroxad)	Dual action: fungicide + nematicide in crops	Watson and Desaeger, 2019
Environmental behavior	Varies among SDHI compounds depending on structure	High persistence, strong soil adsorption, longer half-life in soils	Wei et al., 2016; Vargas-Pérez et al., 2020

## 3 Environmental fate and persistence of fluopyram

**Figure 2:**
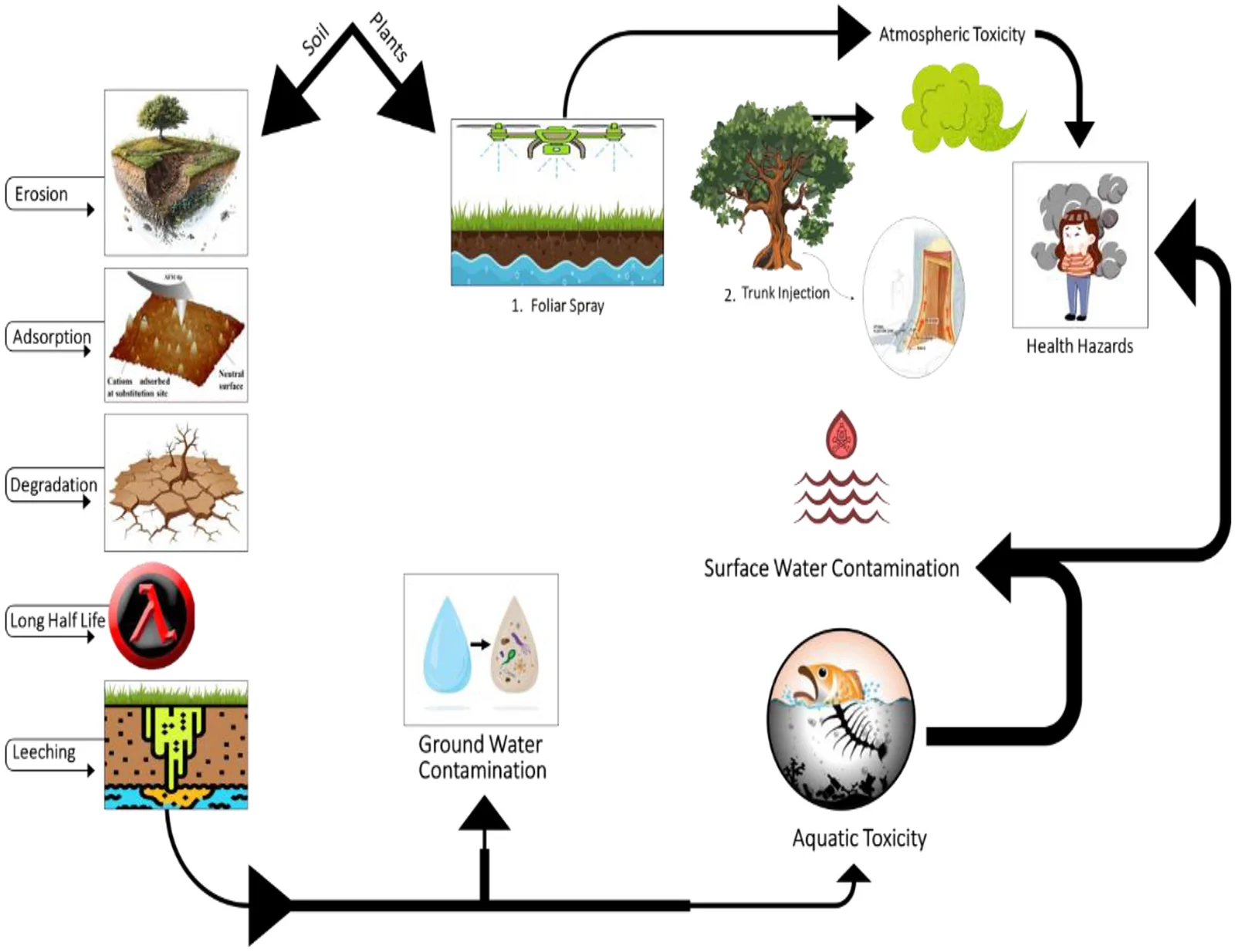
Environmental fate and transport pathway of fluopyram SDHI.

Fluopyram adsorbs strongly to soil particles, and therefore, there is low leach ability and high retention in topsoil, particularly in banana, laterite and red loam soil (Zhou et al., 2021). The adsorption-desorption behavior of fluopyram is strongly dependent on soil properties, which contributes to region-specific patterns of persistence (Table 4). The degradation of fluopyram in soil is a time-dependent and non-classical process, which depends on physicochemical and microbiological characteristics of soil (Paszko et al., 2025). The rate of degradation is higher with the field-capacity moisture than with the air-dry soils, which indicates the significance of soil moisture in dissipation of fluopyram (Lohithaswan et al., 2023). The half-life of Fluopyram has a significant range of variability regarding soil type, environment, and method of application and can vary between a few days and several months (Wei et al., 2016; Zhou et al., 2021). Increased levels of application greatly extend the half-life of fluopyram, and this leads to accumulation in the event of intensive agricultural application (Rathod et al., 2022). Even though fluopyram has generally low leaching as it is well adsorbed to the soil, predictive models suggest that it may move downwards in specific soil and moisture condition (Paszko et al., 2025). Treatment of fluopyram in wastewater with ozone-microbubble has proved to be effective in degrading the pollutant and thus reducing environmental load, but its transformation products are toxic at different levels (Li et al., 2020). Rotational crops may absorb fluopyram residues like scallions in contaminated soils, which means that they may transfer the contaminant to food chains (Yun and Choi, 2023). The volatility of fluopyram is low and will not experience substantial volatilization and long-range transport by the atmosphere. The loss of applied fluopyram to the atmosphere is only a small proportion and the environmental reactions are the most significant determinant of its persistence (Figure 2) (Qanungo et al., 2023).

**Table 4:** Key environmental processes influencing the fate and persistence of fluopyram.

Aspect	Key findings	References
Soil adsorption and retention	Fluopyram is strongly adsorbed to soil particles and is not readily leached and appears to have high levels of retention in the topsoil, especially on banana plantation, laterite and red loam soils.	Zhou et al., 2021
Influence of soil properties	Soil properties will influence the adsorption, degradation persistence will differ with soil chemical-physical and microbiological properties and can result in different environmental fate on a regional level.	Paszko et al., 2025
Persistence and half-life	Half-life and persistence fluopyram persistence varies widely (DAFs-days after application; half-life- amount of time for 50% total degradation in the environment.	Wei et al., 2016; Rathod et al., 2026
Impact of application rate	An increase in application rates will increase persistence and could facilitate accumulation in intensive farming.	Rathod et al., 2022
Leaching potential	Leaching of fluopyram is low under most soil and moisture conditions, but can occur under certain soils and moisture conditions.	Paszko et al., 2025
Uptake by rotational crops	Absorption by rotational crops Residues of fluopyram are absorbed by rotational crops like scallions, which may be one of the routes to enter into it.	Yun and Choi, 2023

The environmental fate and transport of fluopyram after application as a foliar spray or trunk injection (Figure 2). Once applied, fluopyram can undergo interactions with soil and plants, which are affected by the adsorption, degradation, persistence, erosion, and leaching processes. Fluopyram has a relatively long environmental half-life and can leach into groundwater and run-off/erosion into surface waters. Environmental dispersion may result in non-target exposure and contamination in the atmosphere. Fluopyram residues in aquatic systems may affect aquatic life, and exposure to the environment can create potential risks to human health and/or ecological effects (Lewis et al., 2016; EFSA (European Food Safety Authority), 2013).

## 4 Soil behavior

### 4.1 Adsorption

Physicochemical properties of fluopyram are the moderate water solubility and lipo philicity, which makes the product moderately mobile in the agricultural soil (Majid et al., 2024). Soil organic matter is notable as far as the control of fluopyram adsorption and retention is concerned (Khan et al., 2023). The behavior of sorption is also changed due to the content of clay and texture of the soil as those determine the number of binding sites and surface area (Nguyen et al., 2025). The sorption process with respect to time occurs since the fluopyram is slowly absorbed into the soil micropores. The desorption process is not as fast as the adsorption, and it leads to the long residence time of soils. Adsorption and desorption being hysteretic indicates that the binding of fluopyram is not fully reversible (Urseler et al., 2022).

### 4.2 Degradation kinetics

Microbial degradation is the primary pathway of dissipation of fluopyram before the use of aerobic soils (Singh et al., 2026). The bacterial and fungal groups complementary work in the fluopyram biotransformation (Zhao et al., 2025). Degradation in the laboratory and the field case is typically of first-order kinetic effect. However, biphasic kinetics may occur also when the fractions strongly sorbed are no longer bioavailable. The soil moisture and temperature are other factors of consideration that determine the degradation rate. The rise in temperature raises the metabolic rate and activity of microbes and dissipation (Nowak et al., 2025). Strong sorption bonds can retard the activity of microbes and degradation. High-occurrence soils with high organic carbon are likely to be subjected to low apparent soils degradation whilst an increase in microbial biomass (Sarker et al., 2024).

### 4.3 Half-life variability

Based on the given soil half-lives, on varying environmental conditions, they are different in terms of weeks and several years (Patel et al., 2025). Half-life variability shows the variability of the climate, soil chemistry, microorganisms activity and type of formulation. The half-life of soils in tropics is low due to the high activity levels of the microbes. High amounts of precipitation as well as high temperatures promote fast dissipation of agroecosystems in tropical areas (da Silva et al., 2024). The subsoil environments are highly tenacious because they lack the oxygen and contain microbes. A drop in the rate of degradation in the deeper horizons can be attributed to the reduced degree of nutrient availability and the concentration of the types of microbial diversity (Salz et al., 2026). Repetitions might cause accumulation in soils that have low degradation capabilities. The buildup is endemic in posing the threat of permanent exposure of soil organisms and plants.

## 5 Water and sediment fate

### 5.1 Leaching potential

Fluopyram exhibits moderate leaching ability particularly in low organic soils of the sandy soils (Alfee and Bloor, 2025). The adsorption capacity of course texture soils is lesser and therefore they can move more downwards as the water that enters the soil permeates. The studies are modeling studies, which rely on the expected overruns of the groundwater threshold under vulnerable circumstances. Simulations are used to suggest that risks are elevated in both deep groundwater table scenarios and low application frequency (Seidl et al., 2022). Field monitoring of field surfaces and groundwater has been done to confirm the use of fluopyram in agriculture areas. Seasonal variation of rainfall also centers its attention on exerting a substantial effect to concentrations of compartments of water observed. Rainfall is one of the largest ways through which the transport to the surface waters is carried out through the runoff. The excessive precipitation following the application could give rise to pulse injections into the neighboring streams (Fong et al., 2020).

### 5.2 Water pollution surface and groundwater

Surface water bodies in areas with high agricultural activity often contain pesticide residues, such as SDHI fungicides like fluopyram, due to runoff, leaching, and frequent application (Navarro et al., 2024). The frequency of fungi application is also high in the location of detection (Bharani et al., 2024). Fluopyram has low hydrolysis and photodegradation rates in water bodies, enhancing its persistence. Abiotic degradation is also hampered in low light penetration conditions and neutral pH whereas sluggish anaerobic degradation enhances its build-up in sediments, which serve as long-term stores (Chen et al., 2022). Sediment organic matter has a significant impact on the level of sorption and persistence. Re-suspending the contaminated sediments, re-distributes fluopyram into the water column increasing environmental half-life.

## 6 Atmospheric transport

### 6.1 Volatilization and drift

Fluopyram is less volatile leading to the minimization of volatilization losses (Hung et al., 2022). Long-range transport (through the atmosphere) of the contaminants is non-significant compared to the soil and water-based transport (Mehdizadeh et al., 2025). The most common path of exposure to the atmosphere is the spray drift in the course of application. Drift can cause the deposition of off targets on other ecosystems. The comparative environmental persistence and degradation behavior of fluopyram across different environmental matrices (Table 5).

**Table 5:** Environmental half-lives across matrices.

Matrix	Half life	Conditions	References
Water–sediment	>1,000 days	Anaerobic sediment	Sarker et al., 2024
Surface water	21–25 days	Photolysis	Feist and Lance, 2021
Soil (tropical)	36–58 days	Warm, high microbial activity	Luarte et al., 2023
Soil (subsoil)	>1,000 days	Low oxygen, low microbes	Guo et al., 2025
Soil (temperate topsoil)	207–887 days	Moderate climate	Jespersen et al., 2024

## 7 Plant metabolism

### 7.1 Primary metabolites

When foliar material or soil is applied to plant foliage or soils, the fluopyram enters through tissues of plants, and is broken down (Authority et al., 2020). The hydroxylation and cleavage of benzamide moiety is the important plant metabolite pathway. Fluopyram-benzamide and fluopyram-hydroxylated were reported to be prominent identified plant metabolites (Lu et al., 2025). The conjugation reactions involve the glucosylation and malonylation involving phase I metabolism. Such conjugates are biologically inactive as compared to the parent compound (Lee et al., 2013).

### 7.2 Eating things leftover/remnant action

The major components that store the residues of the fluopyram, which is an application-dependent compound, are fruits, roots, and leaves (Pan et al., 2025). Translocation Systemic translocation may be applied when treating surfaces and moving them to inner plant tissue (Authority et al., 2020). The amounts of residues in eatable parts go down over time, by metabolism and dilution of growth. The level of residues is further reduced through washing and cooking subsequent to harvesting. However, even the residues of low concentration may remain on crops until harvesting (Kang et al., 2024).

## 8 Soil and microbial metabolism

### 8.1 Microbial degradation pathways

The key consideration when it comes to biotransformation of the fluopyram is the microorganisms that are present in the soil (Singh et al., 2020). Applied microbial reaction biodegradation The first one is the amide hydrolysis reaction and oxidation with the help of appropriate enzymes of the microorganism (Dong et al., 2024). The bacteria and fungi destroy fluopyram in the aerobic environment. On the other hand, anaerobic degradation rate is significantly slower due to few enzyme routes. Microorganisms Biomaterials play a major role in the process of degradation rates and paths (Zhang et al., 2024).

### 8.2 Stable vs. toxic metabolites

A portion of the metabolites of fluopyram is mineralized rapidly as CO_2_ and water. Other transformation products have a greater persistence than the parent molecule does (Authority et al., 2020). The metabolites may be fungicidal or biological some of which may be possible sources of chronic exposure. The toxicity of the metabolites varies with the molecular structure and the environmental compartment. Risk assessment is relying increasingly on metabolite profiles; not on the concentration of parent compounds (Paiu et al., 2025). The spectrum of fluopyram metabolites, together with their evaluated toxicity profiles, is summarized (Table 6).

**Table 6:** Identified metabolites and their toxicity profiles.

Metabolite	Matrix	Toxicity profile	References
Fluopyram-glucoside	Plant	Conjugated metabolite with low bioactivity; rapidly excreted	Echeverri-Jaramillo et al., 2020
Fluopyram-sulfate	Animal	Major excretion metabolite with low toxicological concern	Dehghani et al., 2022
Fluopyram-N-dealkylated	Animal and Soil	Possible sublethal effects on soil microbes; low mammalian toxicity	Nowak et al., 2025
Fluopyram-malonylated	Plant	Low toxicity with minor persistence in plant tissues	Chen et al., 2025

## 9 Animal and human metabolism

### 9.1 Liver metabolism

One of the primary metabolism processes within the organism is through which fluopyram is oxidized using cytochrome P450 in the liver (Zhen et al., 2022). The reactions which occur in Phase-I include hydroxylation, de-alkylation and amide cleavage. In phase-II metabolism there is conjugation with glucuronic acid and sulfate. Such metabolism processes render it more water soluble and excrete it more via kidneys and bile. The metabolism rates vary the species due to the difference in their expression of different enzymes (Zhao et al., 2020).

### 9.2 Significant biomarkers of exposure

**Figure 3:**
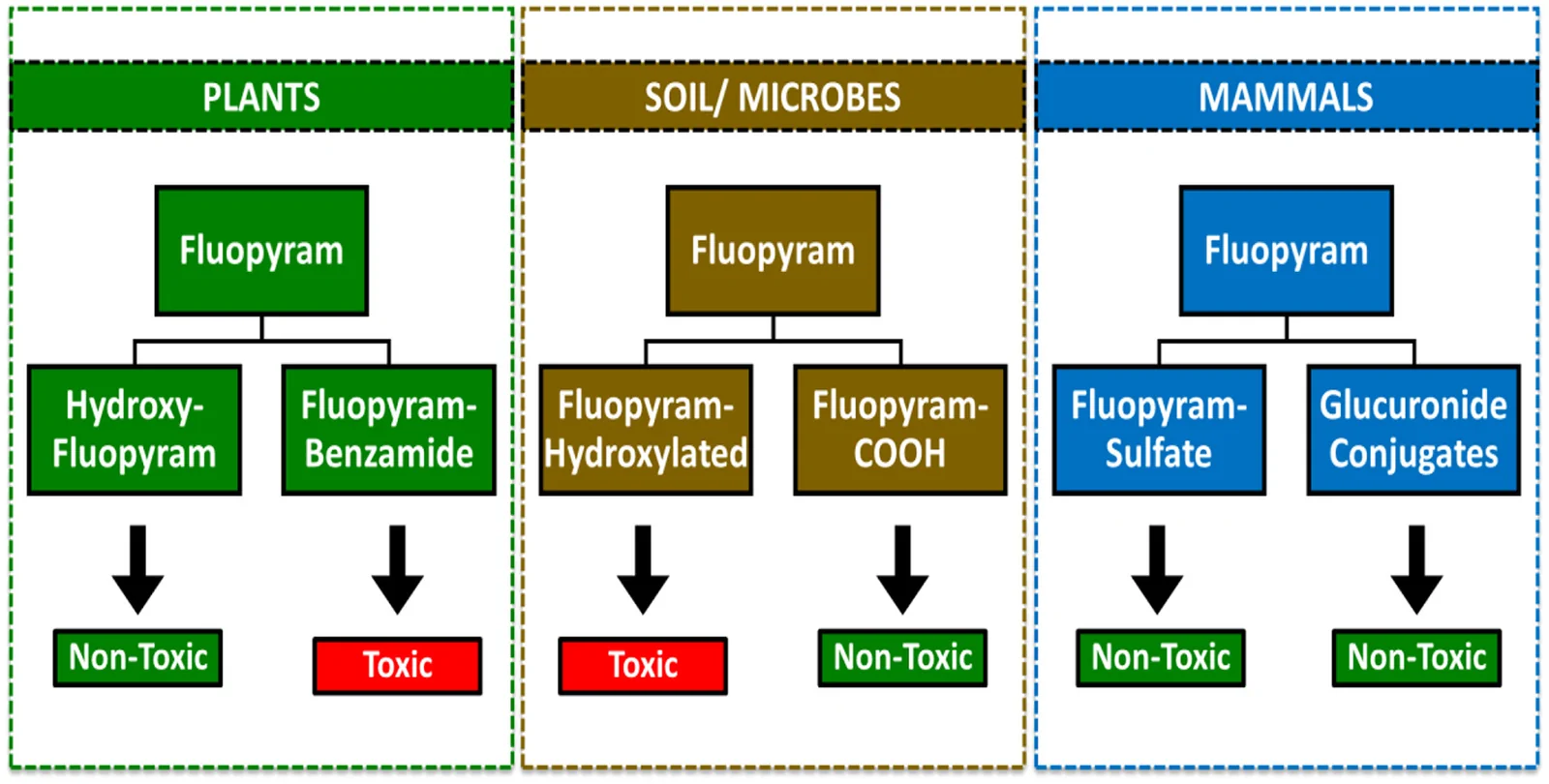
Metabolic pathway in plants, soil, and mammals.

The major exposure biomarkers are hydroxyl-fluopyram and fluopyram-benzamide that are usually urinary metabolites (Klein et al., 2025). Parent compound is effectively evaluated by way of blood plasma measurements as concerns acute exposure. A fat and hair analysis may be a sign of a long-term or cumulative exposure. Exposure profiling through metabolomics is actively embraced in the biomonitoring research (Scholten et al., 2022). These biomarkers are in favor of epidemiological studies which show that there is an outcome of pesticide exposure. The integrated metabolic pathways of fluopyram across plant, soil organisms, and mammals (Figure 3).

### 9.3 Mechanisms of major transformation pathways of fluopyram metabolites

In plants, soil and animals, primary transformation of fluopyram primarily takes place as phase I metabolism including hydroxylation, N-dealkylation and amid bond cleavage via microbial enzymes and cytochrome P450 systems (Mekonnen, 2019; Zhen et al., 2022). Phase II reactions in plants such as malonylation and glucosylation are reactions which produce more polar and less toxic forms of metabolites, by assisting with sequestering and detoxification (Stránská et al., 2025). Under soil conditions, microbial degradation is the primary process and aerobic bacteria/fungi gradually metabolize fluopyram or transform it into persistent intermediate metabolites (Zhou et al., 2021). In mammals, hepatic metabolism results in oxidation and/or another conjugation reaction occurs, resulting in the formation of water-soluble metabolites, which are excreted predominantly as bile and urine (Zhao et al., 2020).

The major metabolic transformation pathways of fluopyram in plants, soil microorganisms, and mammals (Figure 3). In plants, fluopyram is metabolized to hydroxy-fluopyram and fluopyram-benzamide (M25), with M25 representing a toxicologically relevant metabolite. In soil, microbial degradation involves hydroxylation and cleavage reactions, producing hydroxylated and carboxylic acid (COOH) metabolites that influence environmental persistence and mobility. In mammals, fluopyram undergoes extensive biotransformation, including conjugation reactions that generate sulfate and glucuronide metabolites, facilitating detoxification and excretion. These metabolic processes determine the environmental fate, persistence, bioavailability, and toxicological significance of fluopyram across biological systems (European Food Safety Authority (EFSA) et al., 2020; EFSA (European Food Safety Authority), 2023).

## 10 Toxicological profile

### 10.1 Mammalian toxicity

Fluopyram is nontoxic to mammals in low or medium acute orally. The oral LD50 of rats is >2,000 mg kg^−1^ and consequently it has low acute hazard. Systemic toxicity to the skin is also not high since it is not much that is absorbed by the skin and the body has a way of getting it in a short time. The most prevalent form of exposure is occupational inhalation; and it depends on the formulation and mode of application (Martins et al., 2018). Respiratory irritation has only been observed in very high concentrations of aerosols in the air. Experimental animals are mostly affected by the liver and thyroid after repeated exposure (Sood, 2025). Hepatic enzyme induction is a metabolic adaptive mechanism. Chronic exposure to it causes liver weight gain and histopathological changes, which are mild (Authority et al., 2020). It more or less accumulates in the organism due to its lipophilicity (Nguyen et al., 2024).

### 10.2 NOAEL and LOAEL values

**Figure 4:**
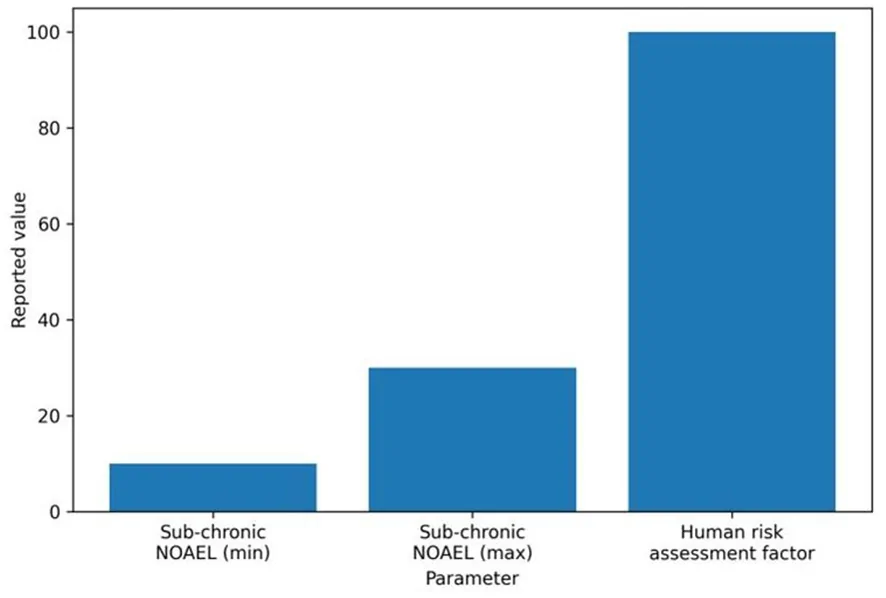
Sub-chronic NOAEL values and the human risk assessment factor for fluopyram.

The primary endpoint values on which NOAEL (no observed adverse effect level) is based are the liver and thyroid (Figure 4). Normal sub-chronic NOAELs range between 10 mg kg^−1^ day^−1^-30 mg kg^−1^ day^−1^ in animals. LOAELs (lowest observed adverse effect levels) are associated with enlargement of the hepatocellular and enzyme amplification. The chronic NOAELs are reduced by the accumulating effects of exposure. The variability between species can be explained by the difference in the rate of metabolism (Norin and Metcalfe, 2019). The regulating authorities apply a human risk assessment factor of 100 and above (Patel et al., 2020). With sensitive populations it is possible to apply other factors of uncertainty. The use of the benchmark dose modeling is gaining popularity over the classical NOAEL (Boots et al., 2021). These agents exert effects on the endocrine system resulting in serious complications (Rouquié et al., 2014).

### 10.3 Inhibition of SDH implications

Fluopyram inhibits succinate dehydrogenase which is key enzyme in mitochondria (Bouillaud, 2023). The SDH inhibition disrupts the tricarboxyllic acid cycle and electron transport chain. It leads to a reduction in the production of ATP and an increase in oxidative stress (Ferrante et al., 2022). There is the possibility of endocrine signaling pathways being impaired due to the malfunctioning of mitochondria. The indirect effect could be on the hormone synthesis that is dependent on energy (Meli et al., 2020). SDH inhibition has caused endocrine-disrupting potential (Authority et al., 2020). Resistance to SDH-inhibitors such as fluopyram can develop in fungi mainly through mutations of the succinate dehydrogenase (SDH) complex that reduce the fungicide binding efficiency but maintain enzyme function (Laléve et al., 2014). In all, resistance arises from a combination of target site mutations, metabolic adaptation and selection pressure, highlighting the importance of integrated fungicide resistance management strategies (Zhang et al., 2026). Specifically, amino acid substitutions at critical residues such as H272, P225 and N230 in the SDH B subunit are directly involved in the inhibitor binding sites and lead to reduced sensitivity to SDHI fungicides (Laléve et al., 2014). For SDHI fungicides, resistance management will be based on the use of fungicide rotation and reducing the selection pressure on fungal populations by managing interactions between different modes of action (Fernández-Ortuão et al., 2017). A mixture of the SDHIs with another group of fungicides (e.g., QoIs or DMIs) is recommended to slow the onset of resistance and enhance disease control effectiveness (FRAC, 2021). The timing in the prevention of sub-lethal exposure that contributes to quicker resistance evolution, such as with repeated or excessive doses, is critical if an application reduction is warranted (Andreazza et al., 2021). Other IPM practices such as cultural control and biological control help to minimize use of chemicals (Sharma, 2023).

## 11 Eco-toxicity

### 11.1 Pollinators (bees)

Adult honeybees are not very sensitive to depo-fluopyram. The Oral LD50 is above 100 μg bee^−^^1^. The sublethal effects of it on foraging behavior have been mentioned (Flórez-Martínez et al., 2026). The issue of navigation might affect the functionality of the colonies. The risk is astronomical since prolonged exposure via pollen and nectar causes it (Huang et al., 2025). The sensitive larval stages of the insects can also be considered. Synergistic toxicity of neonicotinoids has been reported (Ige et al., 2025). Risk assessment of pollinators has chronic and sub lethal endpoints. An integrated overview of toxicity endpoints across key non target taxa, including pollinators, aquatic organisms, soil fauna, mammals (Table 7).

**Table 7:** Toxicity endpoints across species.

Species	Exposure	Endpoint	Value	References
Rat	Oral	LD50	>2,000 mg/kg	EFSA Panel on Contaminants in the Food Chain (CONTAM) et al., 2020
Soil Microbes	Soil	Growth inhibition	< 10%	Zhang et al., 2026
Earthworm	Soil	LC50	>1,000 mg/kg soil	Weitekamp et al., 2025
Honeybee	Oral	LD50	>100 μg/bee	Olker et al., 2022
Rabbit	Dermal	NOAEL	25mg/kg/day	Bejarano et al., 2023
Daphnia magna	Water	EC50	>1 mg/L	Peterson, 2025
Mouse	Oral	LOAEL	50 mg/kg/day	Johnson et al., 2022
Fish (Rainbow Trout)	Water	LC50	>2 mg/L	Rico et al., 2025

### 11.2 Aquatic organisms

Fluopyram is not highly toxic to invertebrates in aquatic environment. Daphnia magna undergoes poor reproduction when under chronic treatment (Baker and Meyer, 2024). Sublethal levels of growth and development of fish are influenced. Photosynthetic activity in algae may reduce. Bioaccumulation can encourage trophic transfer (Gimeno et al., 2024). The combination of herbicides and insecticides is more dangerous. Small streams in the agricultural areas are literally vulnerable. The chronic risk in aquatic systems is normally higher than the acute risk (Authority et al., 2020).

### 11.3 . S-FAuna (earthworms, microbes)

As a rule, the earthworm is not very acutely toxic. The deadly impacts are less than the reproduction ones (Kniuipyte et al., 2025). An inhibition of the microbial respiration of the soil is possible. The structure of the microbial communities may be altered (Sharma et al., 2026). Problems may exist with the interference of cycling of nitrogen and carbon. Missile creatures are so sensitive soil fauna, which is an indicator of early warning. The long-term soil exposure raises the concerns of sustainability (Authority et al., 2020). The risk assessments are increasingly becoming linked to endpoints that are related to soil health. The mechanistic toxicity pathways underlying these affects across different organisms (Figure 4).

**Figure 5:**
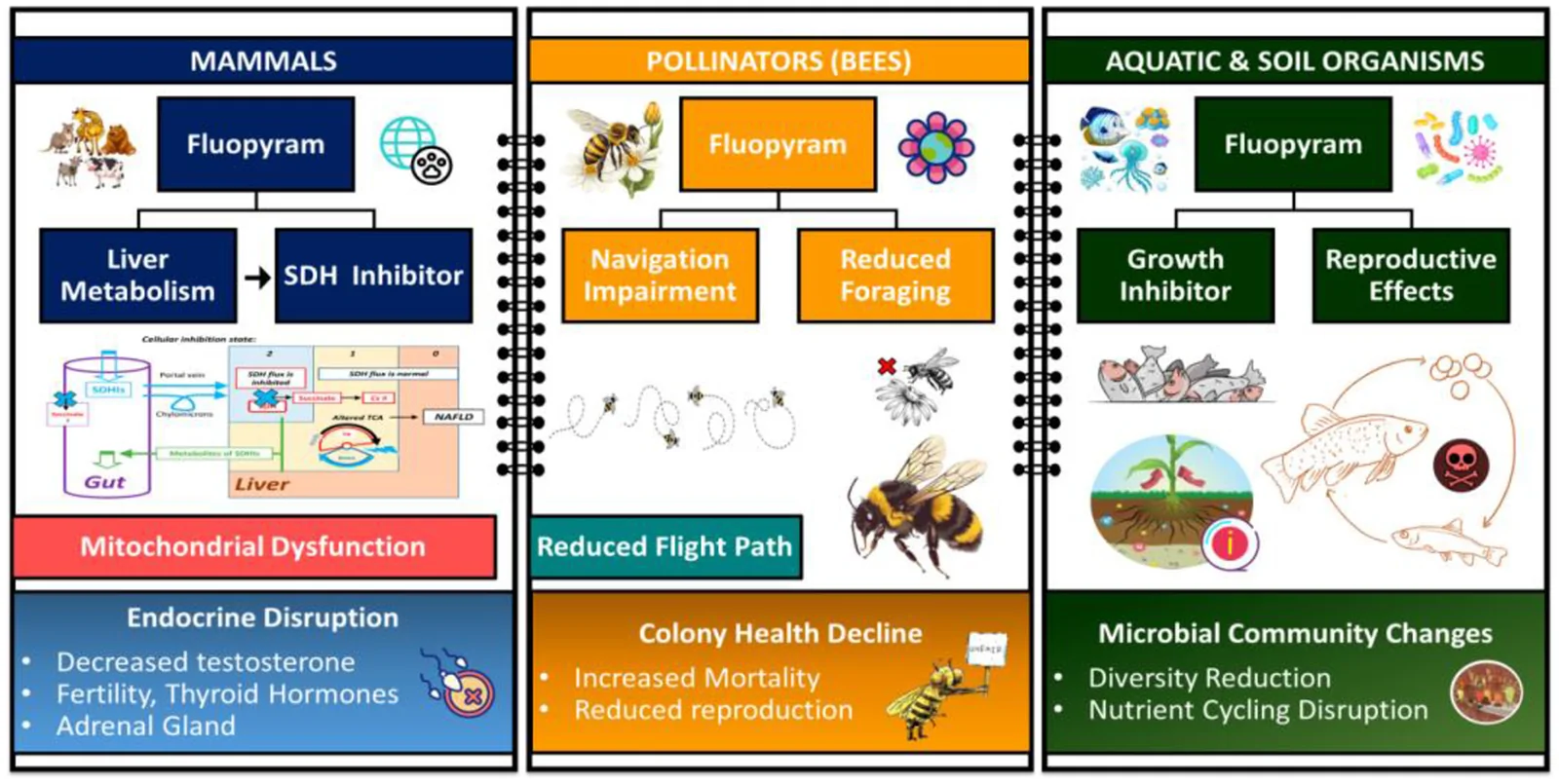
Mechanistic toxicity pathways.

Mechanistic toxicity pathways of fluopyram to mammals, pollinators, aquatic and soil organisms (Figure 5). Fluopyram is a succinate dehydrogenase inhibitor (SDHI) that inhibits the activity of Complex II, which results in reduced mitochondrial respiration and mitochondrial dysfunction. In mammals this disruption may be implicated in metabolic changes of the liver and effects on the endocrine system. Pollinator exposure can negatively affect their ability to navigate, forage, fly, and develop colonies. Fluopyram exposure in aquatic and soil organisms could reduce growth and reproduction rates and impact the microbial community structure, affecting biodiversity and the cycling of nutrients. These effects together show the potential impacts of chronic environmental exposure to fluopyram on the environment and its inhabitants, both ecologically and toxicologically (Bénit et al., 2019; Tosi and Nieh, 2019; Simon-Delso et al., 2015).

## 12 Human and environmental risk assessment

**Figure 6:**
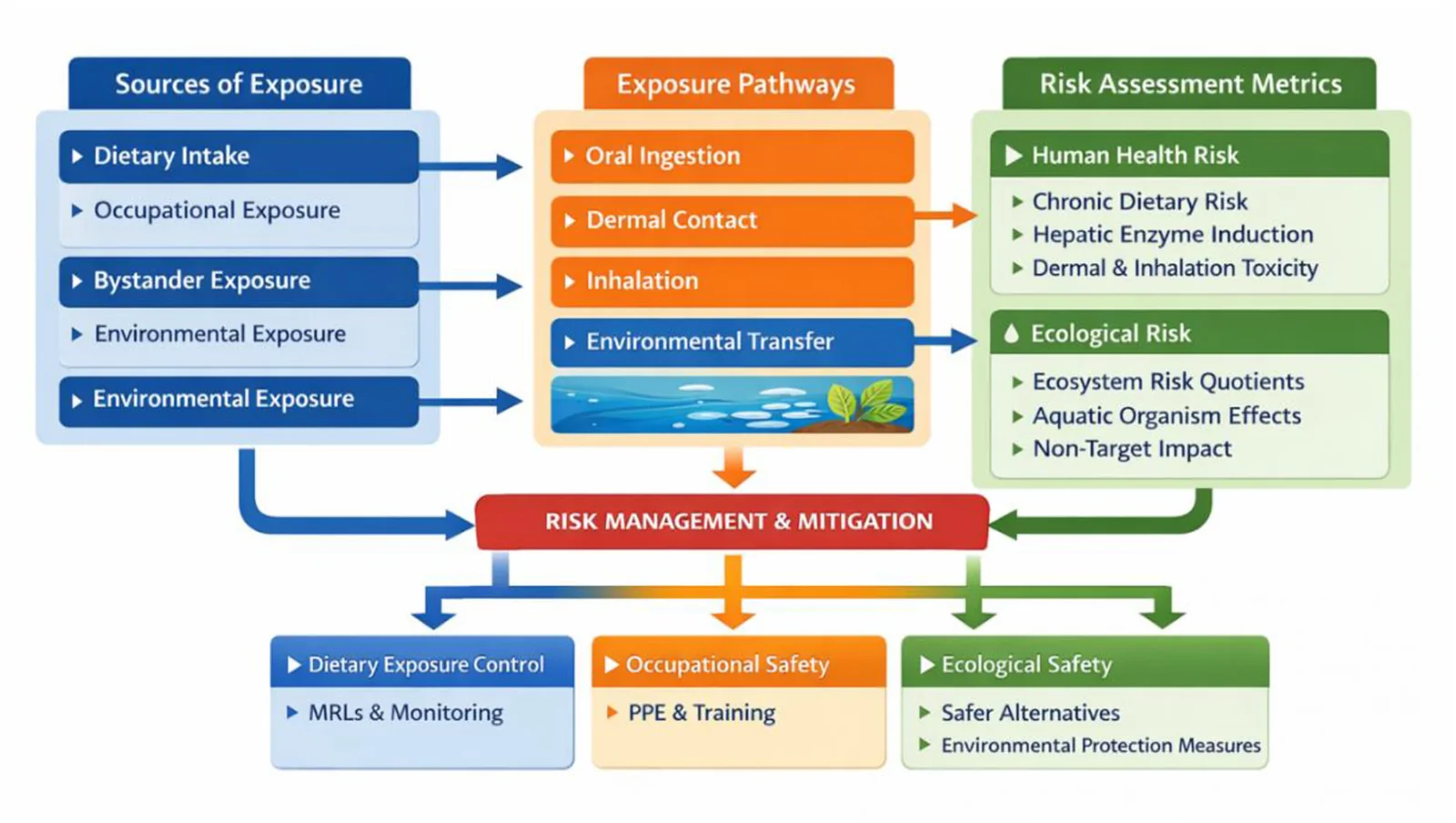
Exposure risk assessment framework.

Residue levels of SDHI pesticides, such as those in strawberries, peaches, and table grapes, were found to be within safe limits for French consumers, indicating no unacceptable chronic risks through diet (Trenteseaux et al., 2024). Most SDHIs induce human hepatic CYP3A4 expression *in vitro*, but environmental or dietary concentrations are generally below levels of concern (Kerhoas et al., 2024). Fluindapyr residues can cause oxidative stress and epidermal damage at high exposure concentrations, highlighting the importance of monitoring dietary intake (Ji et al., 2023). Applicators handling SDHI fungicides, nematicides, or novel pesticides face potential risks if protective measures are not employed (Umetsu and Shirai, 2020). Fluopyram shows toxic effects on non-target organisms and humans if direct contact or inhalation occurs, emphasizing the need for careful handling during application (Bénit et al., 2019; Bouly et al., 2024). The major exposure pathways and risk assessment frame work for fluopyram and related SDHI (Figure 6). SDHI fungicides, including fluopyram and fluxapyroxad, can induce oxidative stress, developmental delays, and organ damage in aquatic organisms like zebrafish, raising concerns about water ecosystem safety (Li et al., 2020; Gioutlakis et al., 2025). Non-fumigant nematicides, such as fluensulfone, fluopyram, and fluazaindolizine, reduce toxicity to non-target organisms while effectively controlling nematodes (Oka, 2020; Shen et al., 2025). Sorghum-based biofumigation and low-risk nematicides effectively manage greenhouse tomato nematodes without harming the ecosystem, showing potential for sustainable pest control practices (Magallanes-Tapia et al., 2025).

The steps for the risk assessment of fluopyram exposure from source identification through to risk mitigation (Figure 6). Exposure can be from dietary consumption of treated commodities, handling of commodities during pesticide application, from being in the proximity of the applicator or through environmental contamination. Fluopyram can enter biological systems via three different routes of exposure: oral, dermal, and inhalation exposure. Risk characterization involves comparing estimated exposure to those that cause toxic effects to humans and the environment, including chronic dietary exposures, chronic occupational exposures, and aquatic and non-target ecological exposures. In order to reduce the risks associated with residues occurring in the environment and to ensure the safe use of pesticides, mitigation measures, in particular the use of personal protective equipment (PPE), applicator training, environmental protection measures and maximum residue limits (MRLs), are implemented [European Food Safety Authority (EFSA) et al., 2020; EFSA (European Food Safety Authority), 2023].

### 12.1 Residue detection and analytical techniques for fluopyram monitoring

Analytical procedures that make use of advanced chromatographic techniques as gas chromatography mass spectrometry (GCMS) and liquid chromatography–tandem mass spectrometry (LCMS/MS) have become indispensable for the reliable analysis of the determination of residues of fluopyram in environmental and food matrices. In these, LCMS/MS is the most common technique because of its high degree of sensitivity, selectivity and ability to quantify fluopyram levels at trace amounts in complex matrices like soil, water, and agricultural commodities (Vargas-Pérez et al., 2020). The multi-residue analytical platforms based on UHPLCMS/MS also allow simultaneous detection of fluopyram and other residues thereby enhancing the analytical efficiency in regulatory monitoring (Wang et al., 2024).

### 12.2 Maximum residue limits (MRLs) and regulatory standards (EU, USA, Codex, China)

Fluopyram MRLs in the EU are specified under Regulation (EC) No. 396/2005, and are product-specific and generally range from 0.01 mg/kg to 50 mg/kg according to the evaluations of residue data in the products and information on crop type (European Commission 2024). The crop-specific tolerances set by the EPA for fluopyram comply with US EPA 2025 (1.0 mg/kg banana; up to 50 mg/kg leafy vegetables). The Codex Alimentarius has International Harmonized Maximum Residue Limits (MXLs) for a number of commodities which are considered as reference commodity standards worldwide for trading and for Food Safety Assessment (FAO/WHO Codex Alimentarius Commission, 2024; Ambrus and Yang, 2016). The MRLs for fluopyram in China are regulated under Chinese National Standard GB 2763-2021, allowing to set the residue limits after considering dietary risk assessment and supervised field trials to ensure consumer safety (Chinese National Standard GB 2763-2021).

### 12.3 Resistance management and integrated pest management (IPM) strategies

Generally, Integrated Pest Management (IPM) in coffee is an approach to control diseases using SDHI fungicides along with other management strategies including crop rotation, resistant cultivars and proper irrigation and fertilization (Oliveira et al., 2021). Biological control agents and soil health management also lower the disease pressure as well as the demand for fungicides, further reducing the disease incidence (Tariq et al., 2020). In general, IPM approaches in orchards permit the use of SDHI fungicides over the long-term and also help maintain disease control in an eco-friendly way (Sharma, 2023).

## 13 Regulatory position and worldviews

The regulatory approval of SDHI fungicides, non-fumigant nematicides like fluopyram and fluensulfone indicates a worldwide trend to compounds with better selectivity and lower non-target toxicity than the conventional nematicides (Desaeger et al., 2020; Oka, 2020). In the European Union, SDHI fungicides were granted approval in 2011–2016 to use as crop protectors, such as fluopyram, even though there is still scientific and popular uncertainty about their long-term toxicity and ecotoxicology (Alex and Patrice Marchand, 2022). In China and other Asian countries, fluopyram and its analogous SDHI-based compounds have been approved because of their anti-nematode and anti-fungal effects and are aligned with the local priorities of protection of crop using high-efficiency products in intensive agricultural systems (Umetsu and Shirai, 2020; Xu et al., 2024). SDHI fungicides and nematicides, despite the regulatory approvals, have been faced with a growing regulatory scrutiny owing to the cumulative effects, low-dose effects, and effects on non-target organisms (Buonsenso, 2025). These arguments are further supported by evidence that some so-called low-risk pesticides, including flupyradifurone, can be disproportionately harmful to vulnerable non-target species, which shows that more comprehensive regulatory frameworks are necessary to conduct the safety evaluation of pesticides (Slunge et al., 2023). Fluopyram has also been identified as a multifactorial replacement of older SDHI molecules, like boscalid, in the SDHI class not only as a nematicide but as a fungicidal (Vitale et al., 2016). Comparative research has revealed that there is no cross-resistance between boscalid and fluopyram in the control of gray mold, indicating that it should be used to regulate resistance by using it (Vitale et al., 2016). The currently emerging SDHI subclasses, such as isoflucypram and novel pyrazole-ketonitrile analogs, have distinct binding modes at the ubiquinone site of succinate dehydrogenase, and may have a better efficacy and possibly better safety profiles (Desbordes et al., 2020; Zhou et al., 2023). Global regulatory differences in approval status and oversight of SDHI fungicide and nematicide across regions are demonstrated (Table 8). This Table 8 highlights that while SDHI fungicides and nematicides are globally approved, the EU exhibits stricter regulatory oversight compared to the USA and Asia, reflecting differences in regulatory philosophy and precautionary approaches.

**Table 8:** Global regulatory status of SDHI fungicides and nematicides.

Region	Regulatory status	Key features	References
European Union (EU)	Approved with restrictions	SDHI fungicides (including fluopyram) approved between 2011–2016; increasing regulatory scrutiny due to toxicity, cumulative exposure, and mixture effects	Buonsenso, 2025
United States (USA)	Approved	Fluopyram and related SDHI nematicides approved under risk–benefit frameworks emphasizing agricultural productivity and mitigation measures	Desaeger et al., 2020; Greco et al., 2024
Asia (China, Japan)	Approved	Approval driven by high efficacy against nematodes and fungi in intensive agriculture systems; focus on food security	Umetsu and Shirai, 2020; Xu et al., 2024

## 14 Conclusion

Field-based long-term monitoring of fluopyram residues in all soil water plant compartments should be performed in future studies and used to better describe how these residues accumulate in the environment. Use of LCMS/MS and other high-resolution techniques in advanced residue analysis should be expanded for giving better detection sensitivity for trace amounts in complex matrices. There is also a need to examine transformation products and metabolites for their toxicity, as they can be important to overall ecological risk assessment. Considering that the disease epidemiology of plant pathogens remains unpredictable, and outcomes of disease are steadily increasingly influenced by non-host factors such as weather, landscape structure, management practice, and associated boletus, there is a need for more focus on soils that support integrated pest management and reduce disease input through the use of reduced fungicide input strategies.

## 15 Research gaps and future research

Even though the efficacy of SDHI fungicides and the newest non-fumigant nematicides like fluopyram, fluensulfone, and cyclobutrifluram has been demonstrated, their ecological effects in the long-term and under field-realistic conditions of exposure have not been well understood (Oka, 2020; Flemming et al., 2025). Although the compounds are intended to decrease the broad-spectrum toxicity relative to conventional fumigants, there is new evidence that SDHI fungicides can cause adverse effects on aquatic organisms, including developmental and organ-specific toxicity in zebra fish, which is a concern when using chronic ecological exposures (Yanicostas and Soussi-Yanicostas, 2021). In turn, it will be essential to focus the future research on long-term, multi-trophic ecological investigation to evaluate cumulative and delayed impacts of SDHI-based nematicides and fungicides in agro ecosystems (Umetsu and Shirai, 2020). However, the environmental effects of application of fluopyram can be minimized by following integrated pest management (IPM) guidelines such as reducing the use of single-mode-of-action compounds (SMC's), application timing, and chemical rotation (Oka, 2020; Umetsu and Shirai, 2020). Different modes of action improve the selectivity of the mixture with nematicides, such as fluensulfone and cyclobutrifluram, and using this mix with fluopyram can largely reduce the selection pressure on soil ecosystems and reduce the accumulation in the environment (Oka, 2020; Flemming et al., 2025). Resistance development is another significant problem that has become a threat to the sustainability of SDHI fungicides and nematicides in the long term (Chen et al., 2020). SDHI compounds, like fluopyram and isofetamid, have also shown reduced sensitivity and non-target sites resistance in field populations of fungal pathogens including *Zymoseptoria tritici* and *Botrytis cinerea* undermining the efficacy of disease control (Amiri et al., 2014). For SDHI fungicides like fluopyram, a big constraint for long-term sustainability is resistance formation, especially applying to a single biochemical pathway (Chen et al., 2020; McCallum et al., 2025). Mutations in succinate dehydrogenase (SDH) subunits, resulting in reduced binding efficiency of fluopyram, have been observed in fungal pathogens such as *Zymoseptoria tritici and Botrytis cinerea*, and shown to reduce efficacy (Amiri et al., 2014; Chen et al., 2020). The accumulation of several sub-lethal biochemical and physiological stress in non-target organisms under chronic exposure, in particular in aquatic life, has been reported, with SDHI believed to cause developmental and organ-specific toxicity (Yanicostas and Soussi-Yanicostas, 2021; Flemming et al., 2025). Although these non-target impacts are not considered as classical resistance, they are an adaptive stress response and metabolic disruption that, in long-term exposure condition, may affect the balance of the ecosystem (Hua et al., 2020; Umetsu and Shirai, 2020). Newer molecules of SDHI, such as cyclobutrifluram and isoflucypram, have modified binding interactions and better efficacy in lower doses; resistance may be further delayed by using them in structured rotation (Desbordes et al., 2020; Liu et al., 2024). The same can now be said about the plant-parasitic nematodes, because repeated use of single-mode-of-action nematicides can hasten the evolution of resistance (Vetrivelakalai et al., 2025). Thus, the next round of studies ought to be conducted on the topic of resistance monitoring, molecular diagnostics, and incorporation of SDHIs into rotation and combination approaches as the part of integrated pest management (IPM) systems (McCallum et al., 2025). The environmental concern about safer and more sustainable pest management methods has led to the emergence of new nematicidal and fungicidal chemistries that are more selective and less damaging to the environment (Hua et al., 2020). Also, new SDHI generation isoflucypram and cyclobutrifluram have their own binding modes and formulation that can improve efficacy with lower doses and can potentially reduce ecological pressure (Desbordes et al., 2020; Liu et al., 2024). The future study ought to focus on formulation innovation, targeted delivery systems, as well as combination methods to achieve a balance between the effectiveness of pest control and the environmental safety and regulatory acceptability (Flemming et al., 2025).
